# *Aspergillus awamori* MH2 as a novel maltobionic acid producer: production optimization and application

**DOI:** 10.1186/s12934-025-02804-y

**Published:** 2025-08-11

**Authors:** Soad M. Yehia, Yousseria M. Shetaia, Ghoson M. Daba, Faten A. Mostafa, Ali A. Ali, Hassaan El-Menoufy

**Affiliations:** 1 Chemistry of Natural and Microbial products Department, National Research Centre, Cairo, Egypt; 2 Department of Microbiology, Faculty of Science, Ain Shams University, Cairo, Egypt; 3 Food Sciences Department, Faculty of Agriculture, Ain Shams University, Cairo, Egypt

**Keywords:** Maltobionic acid, *Aspergillus awamori*, Plackett-Burman design, Central composite design, Agro-industrial wastes

## Abstract

**Background:**

Producing bio-based chemicals using a straightforward and ecologically responsible biotechnological method is intriguing. Maltobionic acid (MBA) is an aldobionic acid obtained from maltose oxidation and is an industrially applied compound. Having antioxidative and antimicrobial, non-toxic, highly water soluble, moisturizing, metal chelating, mildly sour and slightly sweet characteristics.

**Results:**

This study succeeded in utilizing *Aspergillus awamori* as a novel MBA producer. MBA production was improved through two-step statistical factorial designs. Plackett-Burman Design (PBD) investigating the qualitative interaction between eleven factors (maltose, KNO_3_, NaCl, KH_2_PO_4_, K_2_HPO_4_, MgSO_4_, FeSO_4_.7H_2_O, initial pH, temperature, incubation time and rpm) on MBA production causing 1.37- fold increase. Central Composite design (CCD) analyzing the quantitative interaction between the most MBA production affecting factors (temperature, rpm, KH_2_PO_4_, and incubation period) gave a 1.64- improvement in MBA production compared with un-optimized medium. The addition of agro-industrial wastes (AIW) (corn cobs and artichoke leaves) to optimized medium g/l (maltose, 5; KNO_3_, 1; KH_2_PO_4_, 1.0; MgSO_4_, 0.5; FeSO_4_.7(H_2_O), 0.02; pH, 5.00 at 37.5 °C for 9.5 days at 125 rpm) caused 2.41- and 1.97- fold increase in MBA production, respectively in comparison with the initial production conditions. MBA produced by *A. awamori* MH2 exerted an anti-oxidant activity with a ratio of 86% using DPPH scavenging assay.

**Supplementary Information:**

The online version contains supplementary material available at 10.1186/s12934-025-02804-y.

## Introduction

Maltobionic acid (4-o-α-D-glucopyranosyl-D-gluconic acid, MBA) is a member of lactobionic acid (LBA) combination of bionic and aldonic acids in which glucose is α-1,4-bonded to gluconic acid. MBA is an indigestible disaccharide present in honey [1] a new naturally-derived polyhydroxy bionic acid formed by maltose (MA) oxidation. It is a stereoisomer of (LBA) lactobionic acid having, the same molecular weight of 358.30 g mol^− 1^ and a pKa of 3.86 [2], biocompatible, biodegradable but partially indigestible, antioxidative and antimicrobial, non-toxic, highly water soluble, moisturizing [3, 4], metal chelating, mildly sour [1, 5] and slightly sweet [4]. For all these characteristic features MBA was suggested as a potential suitable replacements for LBA.

MBA has various clinically proven medicinal uses, the calcium salt of MBA is currently used in treatment of calcium deficiency in human body [6]. Maltobionic acid can also form a stable salt with inorganic cations, and when bound to Ca to form calcium maltobionate (MBCa), manifests high solubility in water as compared to existing Ca-containing compounds even in pH above 7.0. The rate of Ca absorption from MBCa is significantly higher than that from CaCO_3_ and calcium gluconate (C_12_H_22_CaO_14_) according to accumulation experiments on rats [7]. MBA can easily bind and solubilize minerals [8], increasing especially the absorption of calcium (Ca^2+^), magnesium (Mg^2+^) [1] and oxidized form of iron (Fe^3+^) [1, 5, 9]. Fukami et al. [10] proved that MBA can be utilized as a promising therapeutic agent for improving the intestinal environment.

In contrast to chemical production techniques, biological synthesis of valuable compounds such as aldobionic acids has certain benefits. However, in chemical production of MBA high pressures or high temperatures are frequently used during synthesis. Biological transformations typically occur at lower temperatures and ambient pressures. Furthermore, using costly and dangerous catalysts can be stayed away from. Conclusively, bioproduction techniques for aldobionic acids appear to be more eco-friendly and if there are large product concentrations, production costs can be lower. There aren’t many research on the microbiological synthesis of MBA, although lactose can be converted to LBA by glucose dehydrogenase (GDH). Gram-negative bacteria are the source of all of these GDHs, which are known to quinoprotein GDH (EC 1.1.5.2) with PQQ as a cofactor.

Additionally, several quinoprotein GDHs have the ability to convert maltose into MBA [3]. Thus, a significant upcoming challenge is the rise in the concentrations of the final product by concentrating on enzymatic production and entire cells strategies [11].

Agro-industrial wastes (AIW), one of the most abundant renewable resources in the world, serves as a primary raw material for numerous industries [12]. Fungi have the ability to effectively separate monosaccharides from plant biomass polysaccharides through a variety of sugar catabolic routes [13–15]. These monosaccharides can subsequently be metabolized for growth and energy [16].

To the best of our knowledge, there are no previous reports on the production of aldobionic acids by *Aspergillus awamori* MH2. Therefore, we specially thought to determine whether certain fungal strains might convert MA into MBA by adjusting the culture conditions to increase the chosen fungal strain’s capacity for MBA production. Plackett-Burman design (PBD) was used to analyze the impact of qualitative interaction between a few dietary and physiological components and MBA production, the most important parameters affecting the production process are identified. Afterward, a second statistical factorial design (Central Composite design, CCD) was used to ascertain the ideal parameter concentration needed to produce the highest amount of MBA.

## Materials and methods

### Chemicals

D-Maltose was purchased from Sisco Research Laboratories (India). Maltobionic acid (MBA) was a product of Carbosynth Limited (UK), Potato dextrose agar (PDA) was a product of Biolife Italiana (Italy) and 1-diphenyl-2-picrylhydrazyl (DPPH) was obtained from Sigma-Aldrich (USA). All other chemicals were of analytical reagent grade.

### Fungal strains and cultivation conditions

Fungal strains were donated by National Research Center, Cairo, Egypt and Department of Microbiology, Faculty of Science, Ain Shams University, Cairo, Egypt. *Aspergillus awamori* MH2 was purchased from Assiut University Mycological Centre (AUMC). They were maintained on potato dextrose agar slants at 4 °C and sub-cultured at intervals from 1 to 2 months.

### Preparation of spore suspension

Fungal strains were grown on PDA plates at 30 °C ± 2 for 7 days. The agar plate was washed with 3 ml sterilized 0.1% (w/v) Tween 80 solution [17]. The spore suspension was counted with a hemocytometer under a microscope and used as inoculum for MBA bioproduction. The production medium was inoculated with 10^7^spores /ml.

### Preparation of MBA production screening medium

The production medium was dispensed in 250 ml Erlenmeyer flasks containing 100 ml of the following composition (%, w/v): Maltose,5; KNO_3_,0.4; NaCl,0.05; KH_2_PO_4_,0.13; K_2_HPO_4_,0.04; FeSO_4_.7H_2_O,0.001 and MgSO_4_,0.05. The prepared medium was sterilized in autoclave at 121 °C for 15 min. After cooling, each flask was inoculated with 10^7^ spores /ml and incubated on a rotary shaker at 150 rpm at 30 °C ± 2.

### Quinoprotein glucose dehydrogenase GDH assay

As mentioned by Oh et al. [3] the decrease in absorbance at 530 nm, which was brought on by the enzymatic reduction of 2, 6-dichlorophenol indophenol (DCIP), was utilized to evaluate the maltose-oxidizing activity. After mixing the substrate (1.35 mL) with 150 mL of enzyme solution and containing 0.075 mM DCIP and 5 mM maltose in 100 mM acetate buffer (pH 5.5), the mixture was incubated at 30 °C for 5 min. It was determined that one unit of activity was the quantity of enzyme needed to oxidize 1 µmol of maltose per minute.

### Qualitative detection of MBA via Thin-Layer chromatography (TLC)

According to Kiryu method [18] with modifications after spotting of MBA-contained samples (culture filtrate) on silica gel 60 F-254 aluminum plates. The plates were eluted with a solvent system composed of ethyl acetate: 80% acetic acid: distilled water (3:2:1, by volume) is used. After separation in the TLC jars, the plate was sprayed with 50% (v/v) H_2_SO_4_ and placed in oven at 105 °C for 5 min followed by air drying. MBA was detected as brownish spots on plates (Supplementary Fig. 1).

### Quantitative analysis of calcium maltobionate

As described by Das and Nandi [19] Eriochrome Black T (1 mg) and NH_4_Cl buffer (2 ml) were added on culture filtrate of the five molds with ultimate goal of selecting the most efficient one, which can transform MA to MBA with high yield. All the tested fungal species were cultivated on the production medium at 30 °C ± 2 on shaking incubator at 150 rpm for different incubation periods (3, 4, 5, 6 and 7 days). Determination of final pH was followed by purification and qualitative determination of MBA. Finally, 10 ml of the broth were neutralized to pH 7.0 by Ca(OH)_2_ and filtered. Each filtrate, about 1 mg of Eriochrome Black T and 2 ml of NH_4_Cl buffer were added. The mixture then was titrated with standardized 0.1 M disodium salt of EDTA. The end point was indicated by faint blue color.

## Optimization of *A. awamori* MH2

### MBA production via statistical factorial designs

#### Plackett-Burman design (PBD)

The purpose of the PBD was to investigate the qualitative effect of 11 parameters on MBA production by *A. Awamori* MH2 giving 12 runs. These factors included the following (g/l): MA(A), KNO_3_ (B), NaCl (C), KH_2_PO_4_ (D), K_2_HPO_4_ (E), MgSO_4_ (F), FeSO_4_.7H_2_O (G)and fermentation conditions like initial pH (H), temperature (J), incubation time (K) and rpm (L). Each parameter was tested with high (H) and low (L) concentrations. The response of the design was expressed as the amount of MBA (mg) produced. The success of the design was analyzed via ANOVA.

#### Central composite design (CCD)

According to the data obtained from PBD the quantitative effect of the most promotive parameters on MBA production was analyzed via CCD. (A) Temperature (°C), (B) rpm, (C)KH_2_PO_4_ (g/l), and incubation period (day) (D). Each parameter was investigated with five levels − 2, -1,0, 1, 2 giving 30 runs with six center points. The response of the design was expressed as the amount of MBA (mg) produced. The success of the design was analyzed via ANOVA.

#### The effect of the addition of agro-industrial wastes (AIW) on MBA production

One gram of different AIW (corn cobs (CC), pea peels (Pea P), potato peels (Potato P), artichoke leaves (AL), and molokhia stems (MS)) was added to optimized MBA production medium g/l (maltose, 5; KNO_4_, 1; KH_2_PO4, 1.0; MgSO_4_, 0.5; FeSO_4_.7H_2_O, 0.02; pH, 5.00 at 37.5 °C for 9.5 days 125 rpm). At the end of the fermentation period MBA production was estimated.

#### Assessment of antioxidant activity of MBA produced by *A. awamori* MH2

2,2-diphenyl- 1-picryl-hydrazyl-hydrate (DPPH) assay was used to determine the antioxidant activity of MBA according to the spectrophotometric procedure proposed by Lee et al. [20] using DPPH with a concentration of 0.4 mmol. All measurements were performed in duplicates and absorbance was measured using spectrophotometer at 517 nm.

DPPH scavenging activity %=[(Abs.control- Abs.sample)/Ab.scontrol]×100.

## Results and discussion

### Screening of different fungal species for MBA production

**Table 1:** Screening of different fungal strains for MBA production using different maltose concentrations and for different incubation periods

Incubation Period (day)	3			4			5			6			7		
Maltose Concentration (%)	1	3	5	1	3	5	1	3	5	1	3	5	1	3	5
*Fusarium Y M.*	0	0	0	0	0	0	0	0	0	0	0	0	0	0	0
*Aspergillus awamori MH2*	112.22	110	0	120.24	112.55	62.13	140.28	126.45	106.21	152.34	130.88	98.25	146.36	100.52	60.12
*Aspergillus niger Y M*	0	0	0	0	0	0	0	0	0	0	0	0	0	0	0
*Aspergillus terrus*	0	0	0	0	0	0	0	0	0	0	0	0	0	0	0
*Pencillium chrysogenium*	76.15	80.22	128.25	70.14	100.36	132.26	82.12	110.68	136.28	105.35	89.45	100.15	98.18	70.22	56.11

As shown in the results of Table (1): firstly, the values of MBA production were varied according to the producer strain, and the incubation period. Secondly, of the tested fungal isolates only *A. awamori MH2* and *Penicillium chrysogenum* were able to convert MA into MBA; in addition, the highest MBA production by *A. awamori* MH2 and *Penicillium chrysogenum* (152.34, and 136.28 mg, respectively) was achieved with the most potent producer strain (*A. awamori* MH2*)* after 6 days with 1% maltose. The prolonged incubation period and higher maltose concentration did not have promotive effect on MBA production. Oh et al. [3] and Bieringer et al. [11] mentioned that *Pseudomonas graveolens* and *P. fragi* were ideal MBA producers. Most studies were concerned with the isolation of LBA producers such as *Acinetobacter sp.*, *Pseudomonas spp.*, and *Psychrobacter spp.* [21, 22].

### Optimization of MBA production by *A. awamori* MH2 using PB statistical design

The interaction between the 11 parameters as shown in Table 2 led to a wide variation in MBA production (0-208.41 mg/100 ml). The maximum MBA production was attained with g/l: maltose, 5; KNO_4_, 1; KH_2_PO4, 0.5; MgSO_4_, 0.5; FeSO_4_.7(H_2_O), 0.02; pH, 5.00 at 35 °C for 7 days 200 rpm causing 1.37- fold increase compared with un-optimized medium. The MBA production can be calculated from the following equation:

MBA (mg/100 ml) = + 45.63–12.19 * KNO_3_ + 15.83 * KH_2_PO_4_ -19.14 * K_2_HPO_4_ + 12.16 * FeSO_4_.7H_2_O -22.18 * pH + 40.65 * temperature + 13.56 * incubation time + 17.83 * rpm.

**Fig. 1:**
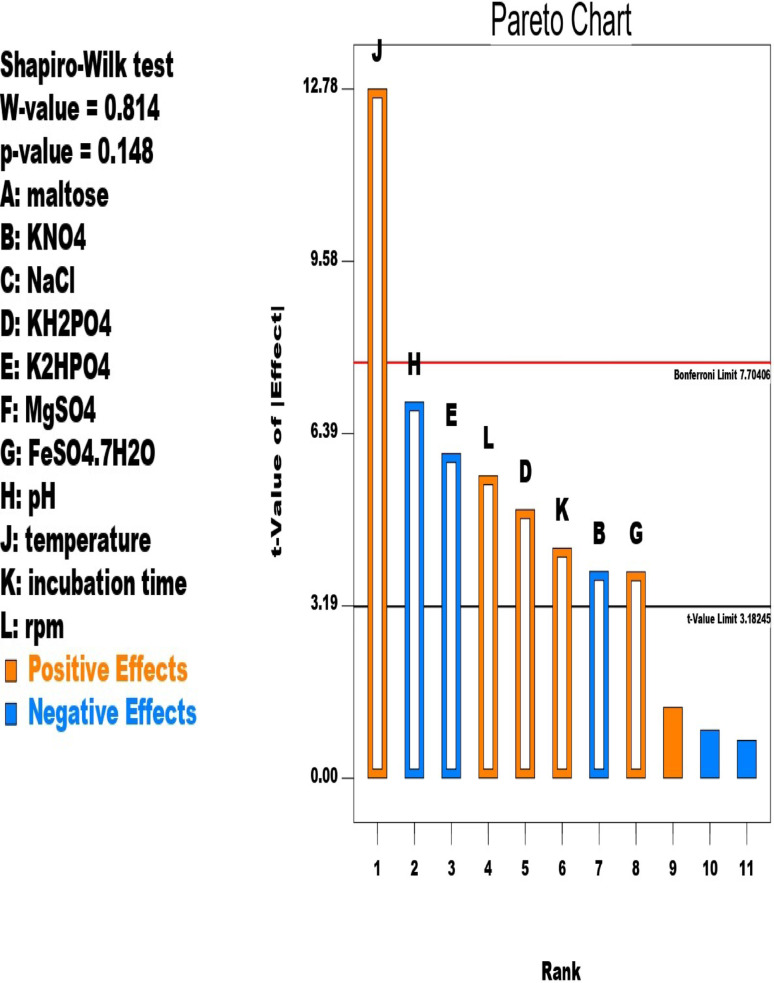
Pareto chart showing the effect of 11 factors on MBA production using *A. awamori* MH2

Pareto chart (Fig. 1) and the equation displayed the parameters that affected MBA production distinguished into those having a positive effect (with + sign) (KH_2_PO_4_, FeSO_4_.7H_2_O, temperature, incubation time, and rpm) and negative effect (with– sign) (KNO_3_, K_2_HPO_4_, and pH) on the production process. As the magnitude before the parameter increased as the most effectiveness of the parameter. El Shayeb and El Minofi [6] studied the effect of different concentrations of, corn steep liquor, urea, and maltose at different pH for various incubation periods on the production of MBA by *Pseudomonas mucidolens* NRRL B-16. It can be concluded that the effect of tested parameters depends on the type of the microbe and the product investigated. KH_2_PO_4_ improved by *Aspergillus awamori* citric acid production [23, 24] while MgSO_4_ reduced its production [23]. Cellulase production was affected by the presence of MgSO_4_.7(H_2_O), CaCl_2_.2(H_2_O), KH_2_PO_4_ and FeSO_4_ 0.7(H_2_O) for *Irpex lacteus*; and MgSO_4_.7(H_2_O), KH_2_PO_4_ and FeSO_4_ 0.7(H_2_O) for *Pycnoporus sanguineus* [25]. KH_2_PO_4_ was sufficient to promote *Ochrobactrum intermedium* growth but not biosurfactant formation [26]. The presence of KH_2_PO_4_ in rapamycin production medium have a positive effect on *Streptomyces* sp. on contrast to its effect on *Streptomyces hygroscopicus* [27].

The success of the design was analyzed through ANOVA (Table 3).

The design is significant, according to its F-value of 43.97. The values of R^2^, adjective R^2^, and predicated R^2^ (0.9915, 0.9690, and 0.8647, respectively) emphasized the effectiveness of the design. i.e. 99.15% of the results can be explained by the design. Values of prob > F were used to examine the significance of the chosen factors; if this value was less than 0.05, it implied the significance of the factors. Implying that KNO_3_, KH_2_PO_4_, K_2_HPO_4_, FeSO_4_.7(H_2_O), pH, temperature, Incubation period, and rpm were significant factors for MBA production by *A. awamori*.

**Table 2:** The recorded response for statistical optimization of MBA production by *A. awamori* MH2 using Plackett Burman design (PBD)

Run	Factor 1	Factor 2	Factor 3	Factor 4	Factor 5	Factor 6	Factor 7	Factor 8	Factor 9	Factor 10	Factor 11	Response 1
	A: maltose	B: KNO_3_	C: NaCl	D: KH_2_PO_4_	E: K_2_HPO_4_	F: MgSO_4_	G: FeSO_4_.7H_2_O	H: pH	J: temperature	K: incubation time	L: rpm	MBA
	g/l	g/l	g/l	g/l	g/l	g/l	g/l		°C	day		mg/100 ml
1	20	1	0.25	0.5	0.5	0	0	5	35	3	200	64.13
2	20	5	0	0	0	0.5	0	7	35	3	200	52.1
3	5	5	0	0.5	0.5	0	0.02	7	35	3	100	36.07
4	20	1	0	0	0.5	0	0.02	7	25	7	200	0
5	5	1	0	0	0	0	0	5	25	3	100	0
6	20	5	0	0.5	0.5	0.5	0	5	25	7	100	0
7	20	1	0.25	0.5	0	0.5	0.02	7	25	3	100	0
8	5	5	0.25	0.5	0	0	0	7	25	7	200	20.04
9	20	5	0.25	0	0	0	0.02	5	35	7	100	60.12
10	5	1	0.25	0	0.5	0.5	0	7	35	7	100	36.07
11	5	1	0	0.5	0	0.5	0.02	5	35	7	200	208.41
12	5	5	0.25	0	0.5	0.5	0.02	5	25	3	200	0

**Table 3:** Statistical analysis of PBD (ANOVA)

Source	Sum of Squares	df	Mean Square	F Value	*p*-value Prob > F	
Model	42718.92	8	5339.865	43.96863	0.0050	significant
B-KNO_3_	1783.397	1	1783.397	14.68455	0.0313	
D-KH_2_PO_4_	3007.383	1	3007.383	24.7629	0.0156	
E-K_2_HPO_4_	4397.989	1	4397.989	36.21319	0.0092	
G-FeSO_4_.7H_2_O	1775.117	1	1775.117	14.61637	0.0315	
H-pH	5904.76	1	5904.76	48.61999	0.0061	
J-temperature	19828.26	1	19828.26	163.2665	0.0010	
K-incubation time	2206.754	1	2206.754	18.17049	0.0237	
L-rpm	3815.263	1	3815.263	31.41501	0.0112	
Residual	364.3415	3	121.4472			
Cor Total	43083.26	11				

### Optimization of MBA production by *A. awamori* MH2 using CCD

**Fig. 2:**
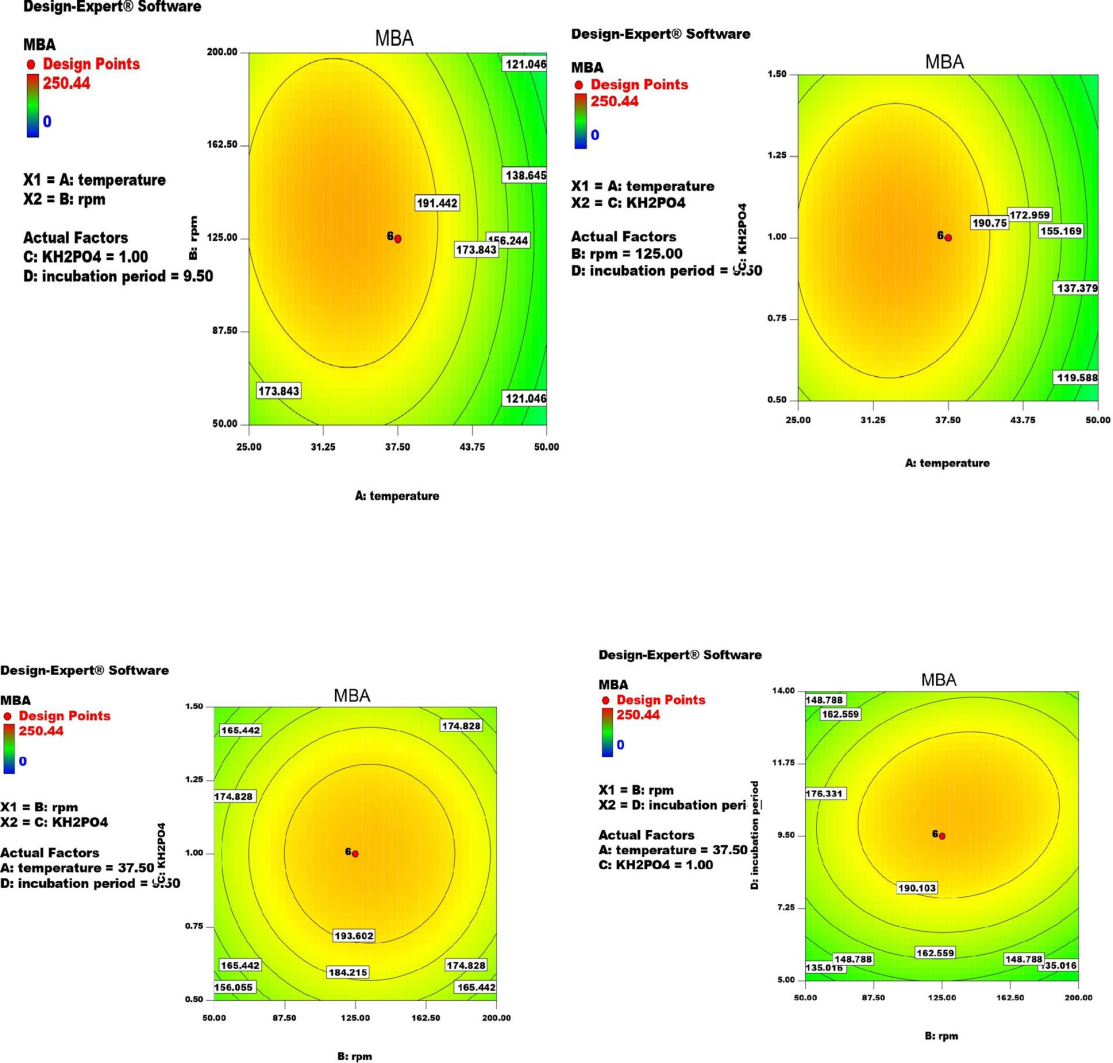

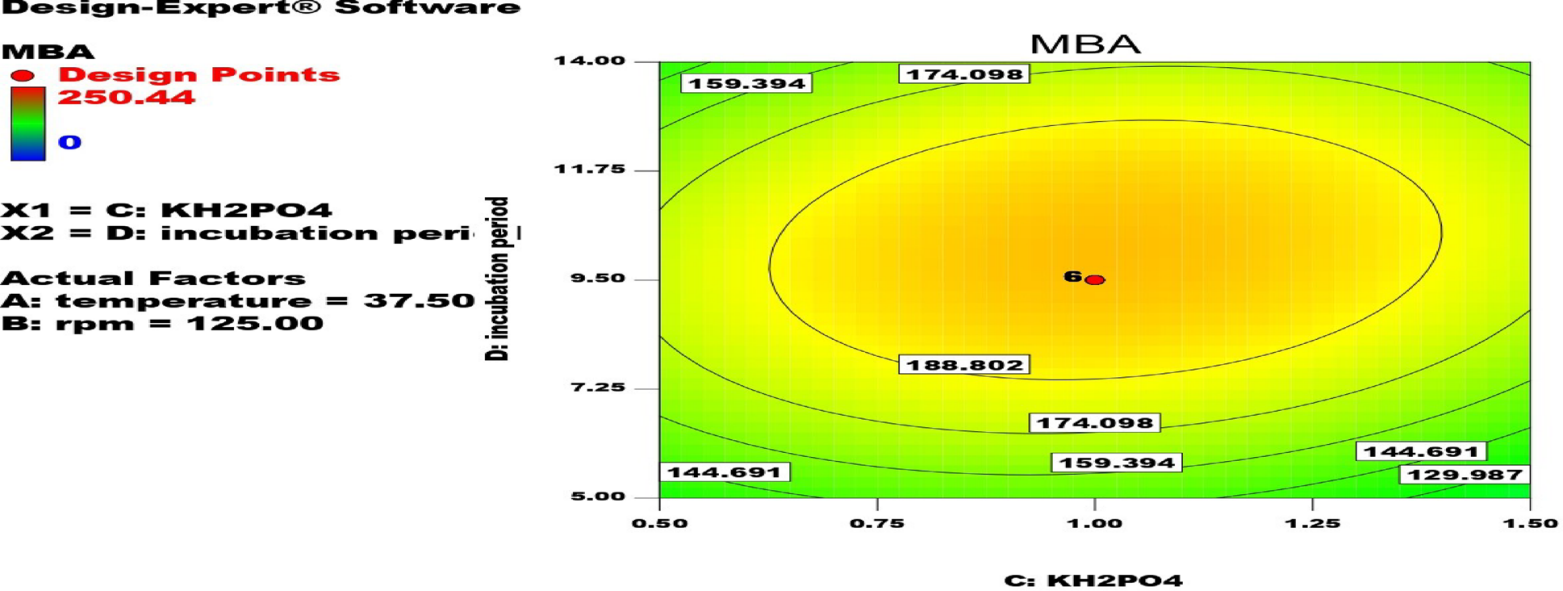
Counter plot showing the interaction between the most effective factors ((A) Temperature (°C), (B) rpm, (C) KH_2_PO_4_ (g/l), and (D) incubation period (day)) on MBA production using *A. awamori* MH2

The quantitative interaction between (A) Temperature (°C), (B) rpm, (C) KH_2_PO_4_ (g/l), and (D) incubation period (day) as shown in Fig. 2; Table 4 led to MBA production improvement by 1.20- and 1.64-fold increase compared with PBD and un-optimized media, respectively. The maximum MBA production by *A. awamori* can be attained with g/l: maltose, 5; KNO_3_, 1; KH_2_PO_4_,1.0; MgSO_4_, 0.5; FeSO_4_.7H_2_O, 0.02; pH, 5; temperature, 37.5 °C; incubation period, 9.5 days and rpm, 125. MBA production can be calculated from the following equation:

MBA (mg/ 100 ml) = + 202.73–31.38 *Temperature + 5.18 *rpm.

+ 0.025 *KH_2_PO_4_ + 11.50 *incubation period − 5.24 * Temperature * rpm + 2.21 *Temperature* KH_2_PO_4_.

-11.35 *Temperature*incubation period.

+ 0.12 *rpm*KH_2_PO_4_ + 7.35 * rpm * incubation period.

+ 8.68 *KH_2_PO_4_*incubation period − 42.19 *Temperature^2^-25.65 *rpm^2^ -25.13 *KH_2_PO_4_^2^ -42.16 * incubation period ^2^.

UMR 5001

**Table 4:** Central composite design for MBA production using *A. awamori* MH2

Run	Factor 1 A: temperature°C	Factor 2 B: rpm°C	Factor 3 C: KH_2_PO_2_ g/l	Factor 4 D: incubation period (day)	Response 1 MBAmg/100 ml
1	25	200	0.5	5	100.15
2	37.5	125	1	0.5	20
3	50	50	0.5	14	22.33
4	37.5	125	1	9.5	172.34
5	37.5	125	1	9.5	192.38
6	37.5	125	0	9.5	100.2
7	25	200	1.5	14	152.3
8	37.5	125	1	9.5	204.41
9	25	200	1.5	5	52.1
10	50	200	1.5	5	20.11
11	62.5	125	1	9.5	0
12	37.5	125	1	18.5	64.13
13	50	200	0.5	5	20.88
14	50	50	0.5	5	40.25
15	50	50	1.5	14	25.98
16	12.5	125	1	9.5	83.93
17	50	50	1.5	5	25.33
18	25	50	1.5	5	65.33
19	50	200	1.5	14	35.65
20	37.5	0	1	9.5	92.18
21	37.5	125	1	9.5	216.43
22	37.5	125	1	9.5	180.36
23	25	50	0.5	14	96.192
24	25	50	0.5	5	90.8
25	37.5	125	2	9.5	120.24
26	25	200	0.5	14	132.26
27	37.5	125	1	9.5	250.44
28	25	50	1.5	14	112.22
29	50	200	0.5	14	25.65
30	37.5	275	1	9.5	124.03

The significance of the design was analyzed via ANOVA (Table 5). The model F-value (19.26) implied the design significance. The values of R^2^ (0.9473), adjective R^2^ (0.8981), and predicated R^2^ (0.8236) emphasized that 94.73% of the results can be explained by the design, the closeness between the values of adjective R^2^ and predicated R^2^ also emphasized the success of the design. A, D, A^2^, B^2^, C^2^, and D^2^ were significant model terms according to the values of prob F were less than 0.050. There are no reported works considering MBA production improvement through statistical factorial design.

**Table 5:** Statistical analysis (ANOVA) of CCD for MBA production using *A.awamori* MH2

Source	Sum of Squares	df	Mean Square	F Value	*p*-value Prob > F	
Model	128767.3	14	9197.665	19.265	< 0.0001	significant
A-temperature	23627.38	1	23627.38	49.48881	< 0.0001	
B-rpm	644.475	1	644.475	1.349887	0.2635	
C-KH_2_PO_4_	0.014406	1	0.014406	3.02E-05	0.9957	
D-incubation period	3171.516	1	3171.516	6.64291	0.0210	
AB	439.6151	1	439.6151	0.920797	0.3525	
AC	78.37561	1	78.37561	0.164162	0.6911	
AD	2060.071	1	2060.071	4.314927	0.0554	
BC	0.233289	1	0.233289	0.000489	0.9827	
BD	864.4776	1	864.4776	1.810694	0.1984	
CD	1206.312	1	1206.312	2.526684	0.1328	
A^2^	48821.01	1	48821.01	102.2582	< 0.0001	
B^2^	18051.85	1	18051.85	37.81057	< 0.0001	
C^2^	17315.4	1	17315.4	36.26803	< 0.0001	
D^2^	48763.17	1	48763.17	102.1371	< 0.0001	
Residual	7161.432	15	477.4288			
Lack of Fit	3163.584	10	316.3584	0.395661	0.9005	not significant
Pure Error	3997.848	5	799.5696			
Cor Total	135928.7	29				

### Improvement of MBA production via supplementation with agro-industrial waste (AIW)

As shown in Table 6 among the AIW (Potato P, AL, CC, MS, Pea P, OP) supplemented to the optimized MBA production medium only AL and CC attained enhancement effect on MBA production (1.19, and 1.46-fold increase, respectively, compared with the optimized production medium (250.44 mg/ 100 ml)). These differences may refer to the saccharide content differences between the fermented AIW according to Mäkelä et al. [28] and Li et al. [29].

**Table 6:** Assesment the effect of addition of different AIW to MBA production medium

AIW	MBA (mg/100 ml)
Control (without waste)	250.44
Pea P	216.75264
Potato P	266.7808
AL	300.1232
MS	200.07936
CC	366.808
OP	233.4176

In nature, the fungus *Aspergillus niger* degrades plant biomass polysaccharides to monomeric sugars. It transports them into its cells, and uses catabolic pathways to convert them into biochemical building blocks and energy. AL is rich with polysaccharide inulin that hydrolyzed into fructose units enters the cell and is phosphorylated to fructose 6-phosphate before entering glycolysis [29]. The pentoses (xylose and arabinose) from xylan hydrolysis are converted through the pentose catabolic pathway (PCP) to D-xylulose-5-phosphate, which enters the pentose phosphate pathway. The ability of different fungi to utilize different AIW depends on their enzyme production system. *A. niger* could utilize MS as a nutrition substrate for the production of cellulase, pectinase and xylanase [30, 31]. *A. wewitschiae* [32] could utilize AL to produce inulinase.

### Anti-oxidant activity of MBA

MBA produced by *A. awamori* according to DPPH scavenging assay showed 86% anti-oxidant activity and this can be attributed to the fact that MBA structure, is a molecule in which glucose is α-1,4-bonded to gluconic acid possessing multiple hydroxyl groups [10, 11], possessing the properties of oligosaccharides. Consequently, MBA is a desired addition to the food sector, enhancing the flavor of fruit and vegetable juices, processed foods, and increased mineral solubilization; additionally, it covers up bad flavors [8].

## Conclusion

This study may be the first to identify *A. awamori* as an MBA producer. PBD and CCD worked together to increase MBA output, which went from 152.34 mg/100 ml to 250.44 mg/100 ml. The MBA increased to 366.81 and 300.12 mg/100 ml, respectively, with the addition of AIW, particularly CC and AL, to the manufacturing medium. The generated MBA shown antioxidant action, indicating its potential use in the food, chemical, pharmaceutical, and cosmetic industries.

## Supplementary Information

Below is the link to the electronic supplementary material.

Supplementary Material 1

## Data Availability

No datasets were generated or analysed during the current study.
