# Supplementary material for: Aspergillus awamori MH2 as a novel maltobionic acid producer: production optimization and application
Source: Microb Cell Fact. 2025 Aug 11;24:181. doi: 10.1186/s12934-025-02804-y (PMC12337431; doi:10.1186/s12934-025-02804-y)
Supplement: Supplementary file 1 — Supplementary Material 1 [file 12934_2025_2804_MOESM1_ESM.doc]

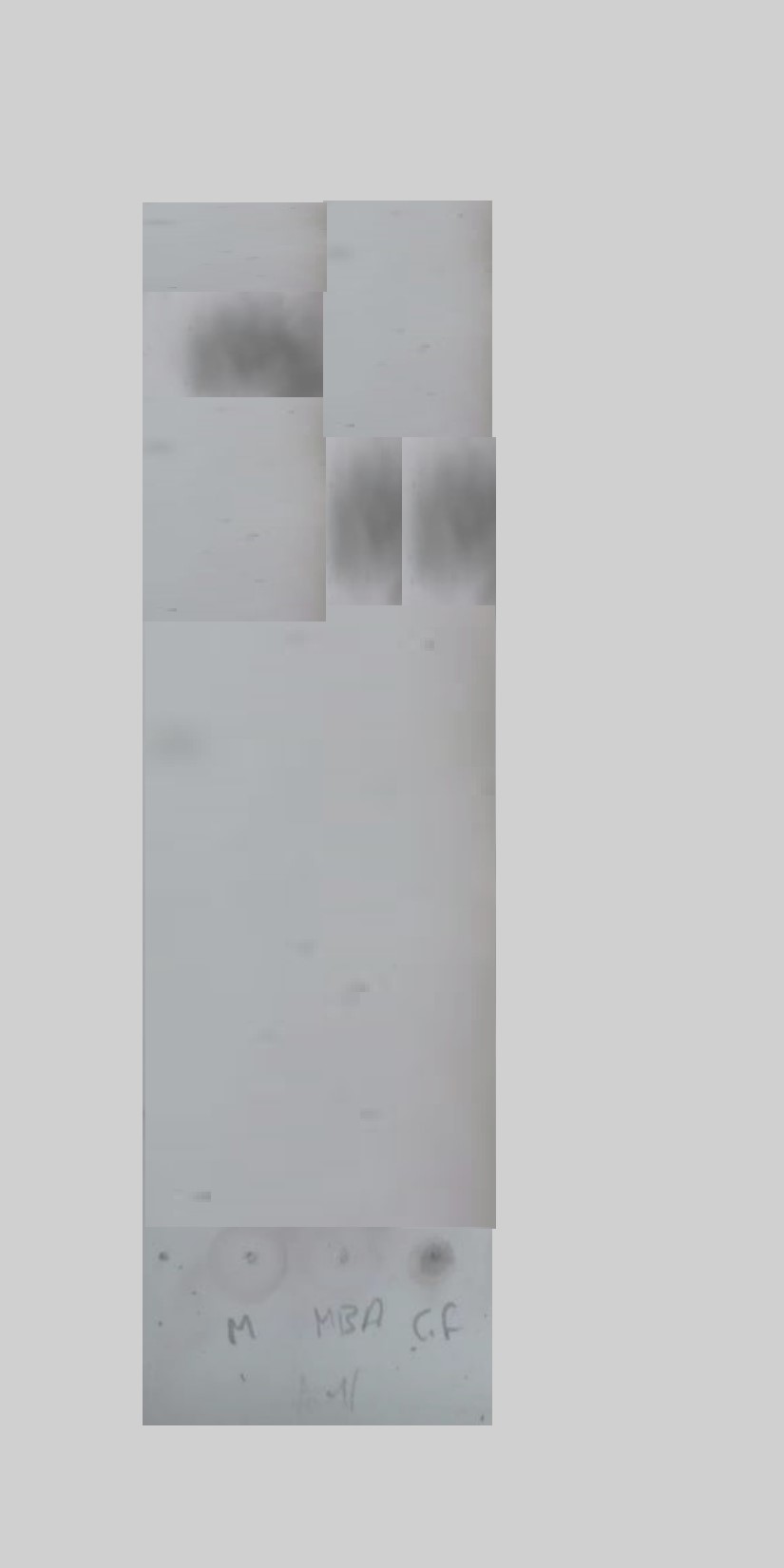


Supplementary Fig. 1: TLC showing the maltose (M) and maltobionic acid (MBA) as markers with *A. awamori* MH2 culture filtrate (c.f.).
